# Global epidemiology of *Giardia duodenalis* infection in cancer patients: a systematic review and meta-analysis

**DOI:** 10.1093/inthealth/ihab026

**Published:** 2021-05-22

**Authors:** Farzad Mahdavi, Alireza Sadrebazzaz, Amir Modarresi Chahardehi, Roya Badali, Mostafa Omidian, Soheil Hassanipour, Ali Asghari

**Affiliations:** Department of Medical Parasitology and Mycology, School of Medicine, Guilan University of Medical Sciences, Rasht, Iran; Razi Vaccine and Serum Research Institute, Agricultural Research, Education and Extension Organization, Mashhad, Iran; Integrative Medicine Cluster, Advanced Medical and Dental Institute, Universiti Sains Malaysia, Bertam, Kepala Batas, 13200, Penang, Malaysia; Department of Microbiology, Faculty of Basic Sciences, Ardabil Branch, Islamic Azad University, Ardabil, Iran; Department of Medical Parasitology and Mycology, School of Medicine, Shiraz University of Medical Sciences, Shiraz, Iran; Gastrointestinal and Liver Diseases Research Center, Guilan University of Medical Sciences, Rasht, Iran; Department of Medical Parasitology and Mycology, School of Medicine, Shiraz University of Medical Sciences, Shiraz, Iran

**Keywords:** cancer patients, *Giardia duodenalis*, *Giardia intestinalis*, *Giardia lamblia*, meta-analysis, odds ratios (ORs), prevalence, systematic review

## Abstract

**Background:**

Application of chemotherapeutics in cancer patients may provide an immunosuppressive milieu, favourable for parasitic infections. *Giardia duodenalis* is an important zoonotic intestinal parasite responsible for diarrhoea in humans worldwide.

**Methods:**

The present systematic review and meta-analysis was conducted to estimate the prevalence of *G. duodenalis* and respective odds ratios (ORs) in cancer patients around the globe. Four online databases—PubMed, Scopus, Web of Science and Google Scholar—were carefully explored for relevant literature without time limitation until 28 November 2020. Meta-analysis was done based on a random effects model to pool the estimations and define 95% confidence intervals (CIs).

**Results:**

The overall weighted prevalence of *G. duodenalis* infection in cancer patients was calculated to be 6.9% (95% CI 0.5 to 9.3) globally, based on data from 32 studies. Although not statistically significant, eight case–control studies revealed that cancer patients were 1.24 times (95% CI 0.66 to 2.31; p=0.501) more exposed to *G. duodenalis* infection than healthy controls. Moreover, the prevalence of infection was not significantly associated with quantitative variables, including publication year (regression coefficient −0.0135, p=0.578), sample size (regression coefficient −0.0007, p=0.074) and human development index (regression coefficient −1.6263, p=0.419). Also, subgroup analysis of the pooled *G. duodenalis* infection was performed for publication year, World Health Organization regions, countries, continents, cancer types and country income.

**Conclusions:**

Altogether, the epidemiology of *G. duodenalis* infection and its associated risk factors in immunocompromised individuals, especially cancer patients, is still open to question and deserves comprehensive investigations.

## Introduction

A quarter of the world suffers from inadequate hygienic settings and diagnostic options, leading to underestimated and/or chronic parasitic infections, which are a major cause of morbidity and mortality worldwide.^1,2^ Such infections are also overlooked in industrialized nations due to their low prevalence and the fact that they do not have pathognomonic signs.^1^ Thus they are a silent threat, particularly in immunocompromised individuals undergoing chemotherapy, leading to hyperinfection by parasitic as well as other infectious agents.^3^ The flagellated diplomonad protozoan *Giardia duodenalis* (also known as *Giardia intestinalis* and *Giardia lamblia*) is the most common species of the genus *Giardia*, infecting various mammals, including domestic animals and humans.^4,5^ In total, epidemiological investigations through 2011 show that approximately 280 million human diarrhoea cases occur annually due to *Giardia* infection, particularly in children <5 y of age, and with a varying prevalence of 0.4–7.5% in developed countries and to 8–30% in underdeveloped countries. Nevertheless, the true prevalence of the parasite is significantly underestimated and much work is needed to accurately clarify this issue.^6–8^

The life cycle of *G. duodenalis* occurs in canine, feline and human hosts. In brief, the parasite encysts in the intestine of susceptible infected humans/animals and the cystic stages are shed to the environment via faeces.^9^ Human infection primarily occurs via the faecal–oral route by consumption of cyst-contaminated food or water and contact with infected hosts.^10,11^ Following excystation by gastric acid and pancreatic enzymes, each cyst releases two motile pear-shaped trophozoites that colonize the duodenum and jejunum and consume bile salts, which further provokes deconjugation and lipid metabolism dysfunction.^12^ In total, disease manifestation depends on the parasite genotype and infective dose as well as host-related factors such as nutritional and immunological status.^13^ Since September 2004, giardiasis was included in the Neglected Diseases Initiatives of the World Health Organization (WHO), due to its negative effect on child health and pregnancy as well as being in parallel with poverty.^11^

The infection is usually asymptomatic. While clinical giardiasis is frequently associated with children <5 y of age or pre-school children living in poor sanitary environments, elderly people and patients with immunodeficiency manifest a variety of gastrointestinal symptoms such as nausea, vomiting, diarrhoea, abdominal cramps and epigastric pain, bloating and progressive weight loss.^14–16^ Notably, chronic *Giardia* infection in children, particularly in developing countries, may be associated with growth retardation and cognitive impairment.^17^ Faecal microscopy is routinely used for the diagnosis of *G. duodenalis* infection. Also, immunodiagnostic assays such as enzyme-linked immunosorbent assay (ELISA) for antibody or copro-antigen detection as well as molecular techniques are applicable.^18^ According to several genetic markers, including small subunit ribosomal RNA (SSU-rRNA) and the triosephosphate isomerase (*tpi*), glutamate dehydrogenase (*gdh*) and β-giardin (*bg*) genes, eight morphologically indistinguishable assemblages of *G. duodenalis* have been confirmed, comprising assemblages A and B (humans and other mammals), C and D (dog and other canids), E (hoofed animals), F (cats), G (rodents) and H (pinnipeds). A single *G. duodenalis* isolate can actually be assigned to different assemblages based on the above-mentioned markers. Identification of the same assemblages or multilocus genotypes in humans and animals of a particular region implicates a zoonotic infection, although the actual role of zoonotic pathways is highly neglected in the epidemiology of giardiasis.^14,19–21^ Humans are mostly infected by assemblages A and B, and to a lesser extent by assemblages C, E and F.^22–24^ Assemblages A and B are further subtyped into AI (mostly zoonotic), AII (mostly anthroponotic), AIII (hoofed animals), BIII and BIV. Convincing evidence suggest that assemblage B is more virulent and prevalent in outbreaks than assemblage A. However, there exists no scientific basis to correlate the course of the infection and/or clinical symptoms to *G. duodenalis* assemblages.^12–14^

More than 2 decades of investigation on *Giardia* pathogenicity indicate that disease initiation and progression is a multifactorial process, being associated with parasitic, host, nutritional, environmental and immunological factors.^25,26^ An in-depth look at *Giardia* pathogenicity shows intestinal barrier dysfunction, elevation of enterocyte apoptosis, host lymphocyte activation, a shortage of brush-border microvilli and atrophy of the intestinal villi, which entails epithelial maldigestion and malabsorption, hypersecretion of anions and subsequent acute diarrhoea. This cascade of events may also facilitate bacterial invasion towards the submucosal layers. Proteomic profiling of *Giardia* trophozoites demonstrated that cysteine proteases, especially cathepsin L (catL)-like and cathepsin B (catB)-like enzymes, may be associated with the increased pathophysiological responses during giardiasis.^27–29^

Diarrhoea is a prominent cause of death in immunocompromised people, with particular emphasis on children <5 y of age. *G. duodenalis* is known as one of the significant agents of diarrhoea in mammals, including humans, along with rotavirus, *Cryptosporidium* species, *Escherichia coli, Clostridium difficile* and *Shigella* species. The disease in people with a healthy immune status is self-limiting, without a clinical course, whereas immunocompromised patients may experience harsh clinical outcomes.^30–32^ Therefore the importance of giardiasis in cancer patients and its proven pathogenicity led us to implement the first global systematic review and meta-analysis on the pooled prevalence of *Giardia* infection and respective odds ratios (ORs) in cancer patients compared with healthy individuals and the associated risk factors.

## Methods

### Systematic search strategy and selection criteria

The results of the present systematic review and meta-analysis were reported based on the Preferred Reporting Items for Systematic Reviews and Meta-Analyses checklist.^33^ Two expert investigators (AA and SH) searched four English electronic databases (PubMed, Scopus, Google Scholar and Web of Science) without a time limitation until 28 November 2020 to retrieve articles investigating the prevalence of *G. duodenalis* in cancer patients globally. For this purpose, the following search keywords were used alone or in combination: ‘intestinal parasites’, ‘parasitic infections’, ‘giardiasis’, ‘*Giardia duodenalis*’, ‘*Giardia intestinalis*’, ‘*Giardia lamblia*’, ‘prevalence’, ‘epidemiology’, ‘frequency’, ‘occurrence’, ‘cancer’, ‘neoplasm’, ‘malignancy’, ‘tumor’, and ‘carcinoma’ using OR and/or AND Boolean operators. A set of keywords was employed for better exploration of relevant literature regarding cancer patients (Table 1). Also, the bibliographies of related papers were scrutinized to extract papers not found through database searching.

**Table 1.** Systematic search strategy in the present study

Databases	Search strategy
PubMed	((((‘Intestinal Diseases, Parasitic’[Mesh]) OR (‘Parasitic Diseases’[Mesh])) OR (‘Giardiasis’[Mesh])) AND ((((Prevalence [Title/Abstract]) OR (Epidemiology [Title/Abstract])) OR (Frequency [Title/Abstract])) OR (Occurrence [Title/Abstract]))) AND (‘Neoplasms’[Mesh])
Scopus	TITLE-ABS (‘Intestinal parasites’ OR ‘Parasitic infections’ OR ‘Giardiasis’ OR ‘*Giardia duodenalis*’ OR ‘*Giardia intestinalis*’ OR ‘*Giardia lamblia*’) AND TITLE-ABS (‘Prevalence’ OR ‘Epidemiology’ OR ‘Frequency’ OR ‘Occurrence’) AND TITLE-ABS (‘Neoplasms’ OR ‘Cancer’ OR ‘Tumor’ OR ‘malignancy’ OR ‘Carcinoma’)
Web of Science	((‘Intestinal parasites’ OR ‘Parasitic infections’ OR ‘Giardiasis’ OR ‘*Giardia duodenalis*’ OR ‘*Giardia intestinalis*’ OR ‘*Giardia lamblia*’) AND (‘Prevalence’ OR ‘Epidemiology’ OR ‘Frequency’ OR ‘Occurrence’) AND (‘Neoplasms’ OR ‘Cancer’ OR ‘Tumor’ OR ‘malignancy’ OR ‘Carcinoma’))
Google Scholar	Using related keywords

Initial screening was only based on the abstract and title of papers. After duplicate removal, the full texts of eligible articles were obtained via online databases. Evaluation of eligibility was done by four trained investigators and possible disagreements were settled by discussion and consensus with the fifth reviewer. The following inclusion criteria were used for qualified studies: the study population was limited to cancer patients; peer-reviewed original papers without any geographical and time limitation until 28 November 2020; cross-sectional studies investigating *G. duodenalis* prevalence in a particular sample size of cancer patients; case–control studies reporting cancer (as exposure) and *G. duodenalis* infection (as outcome) having specified ORs; and molecular- and/or microscopy-based studies evaluating stool samples regarding *G. duodenalis* infection. Those studies that did not meet the inclusion criteria, including case studies, reviews, letters, studies on non-cancerous immunocompromised patients and/or immunocompetent individuals, animal studies, seroprevalence reports, experimentally infected individuals, studies without prevalence reports and studies with unclear/confusing information were excluded from the present review. The following variables were extracted using a predesigned checklist for each study: first author's last name, quality assessment score, publication year, implementation year, continent, country, WHO region, country income, study type, cancer type, total sample size, infected sample size and Human Development Index (HDI). In the present study, information about country income was obtained from the World Bank https://datahelpdesk.worldbank.org, which has been updated through 2019.

### Quality assessment and data extraction

The quality of the papers was another parameter required for the inclusion of relevant records. For this purpose, Joanna Briggs Institute (JBI) Critical Appraisal Checklist for Studies Reporting Prevalence Data was employed.^34^ Those articles that scored 4–6 and 7–10 points were deemed moderately and highly qualified, respectively. Accordingly, articles with a score of ≤3 points were excluded from this systematic review.

### Data synthesis and statistical analysis

Statistical analyses were conducted using the Comprehensive Meta-Analysis version 3 software (Biostat, Englewood, NJ, USA). The prevalence of *G. duodenalis* infection in cancer patients was assessed by computing pooled prevalence and 95% confidence intervals (CIs) using a random effects model. This model is used in the case of heterogeneity, which provides the distribution of true effect sizes among published papers. Subgroup analyses were used to estimate the weighted frequency of *G. duodenalis* infection based on WHO regions, geographical distribution, country incomes, publication years, continents, cancer types and HDI. Weighted odds ratios (WORs) and 95% CIs were calculated to correlate the *G. duodenalis* infection to cancer patients and their respective control groups. Also, any variations in the finally calculated WORs were evaluated by sensitivity analysis. The results were shown as forest plots of the weighted prevalence (with 95% CI) of *G. duodenalis* infection in cancer patients. The funnel plot was used to check the probability of publication bias during the analysis. Meta-regression was used to assess the possible association between variables such as publication year, sample size and HDI index with *G. duodenalis* prevalence in cancer patients. Heterogeneity between studies was assessed using the I^2^ index, so that I^2^ values <25%, 25–50% and >50% were considered to have low, moderate and high heterogeneity, respectively. P-values <0.05 were considered statistically significant.

## Results

### Summary of the systematic search

**Figure 1.**
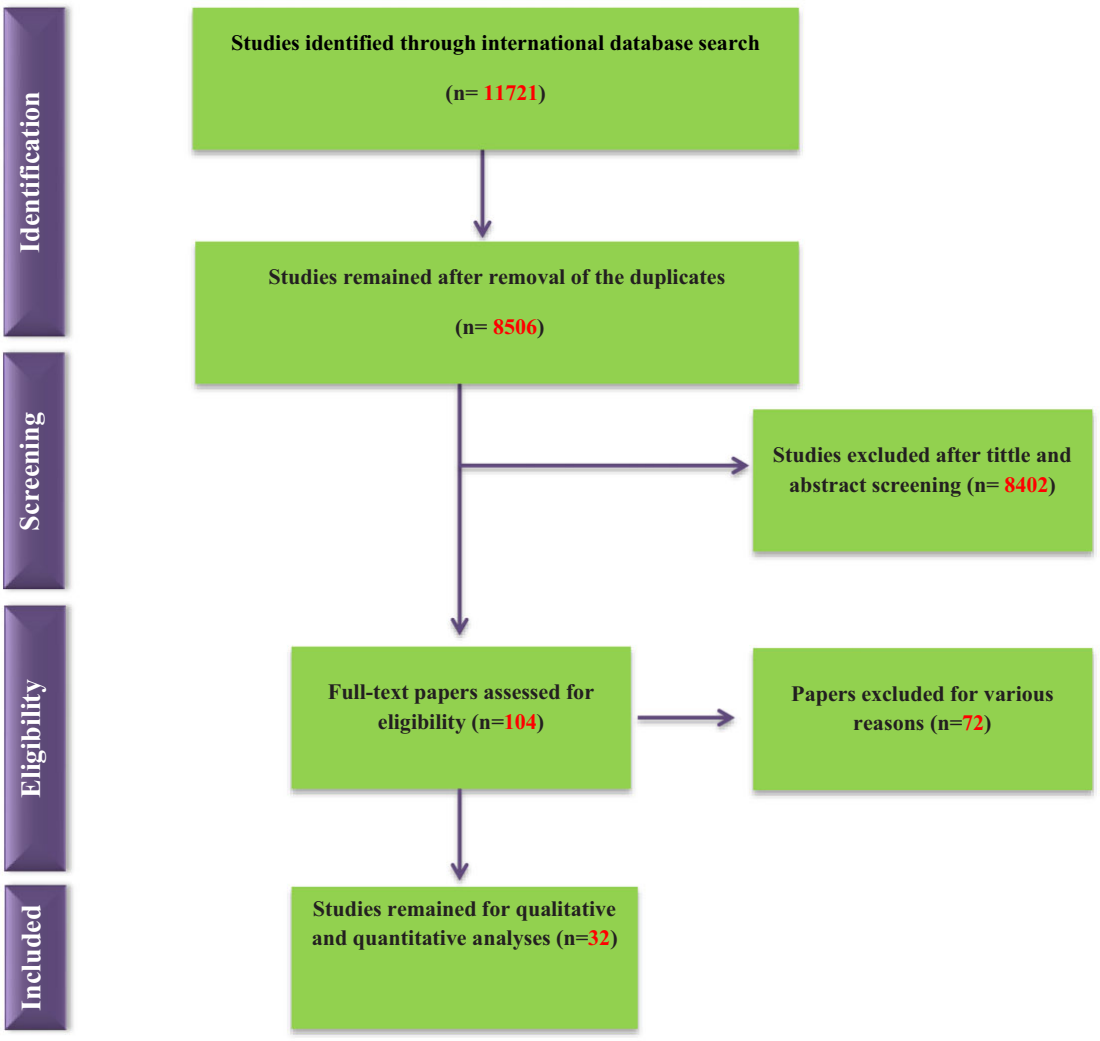
Flowchart of the included eligible studies in the systematic review.

Figure 1 provides a flowchart summarizing the procedure of the systematic search strategy and selection of qualified studies. In brief, our primary systematic searching identified 11 721 papers. After initial screening based on title and abstract along with removal of duplicates, 104 articles were subjected to the complete review process by trained investigators. Of these, 32 papers qualified to be included in the present systematic review and meta-analysis.

### Qualitative and quantitative characteristics of included studies

The main characteristics of the included papers are provided in Table 2. According to geographical location, most studies (14 papers) were from Iran,^35–48^ followed by 3 from Turkey,^49–51^ 3 from Egypt,^52–54^ 2 from Brazil,^55,56^ 2 from India,^57,58^ and 1 each from Indonesia,^59^ Iraq,^60^ Malaysia,^61^ Mexico,^62^ Poland,^63^ Uganda,^64^ Uzbekistan^65^ and Yemen.^66^ Overall, 21 studies were from Asia (3706 individuals), followed by 4 studies from Africa (2268 individuals), 4 from Europe (687 individuals), 2 from South America (143 individuals) and 1 from North America (77 individuals). The sample size ranged from 10 to 1771 individuals and the oldest study was conducted in 1997.^57^ A total of 26 studies were done among patients having mixed cancer types, followed by 4 and 2 studies on patients with haematological malignancies (HMs) and colorectal cancer (CRC), respectively. Based on the epidemiological design of studies, 27 were cross-sectional studies, whereas only 8 had a case–control design. Among all included studies, 27 assessed the *Giardia* infection by faecal microscopy and 5 studies used both microscopic and molecular techniques. The JBI checklist found that 6 articles had high quality (>6 points) and the remaining 26 had moderate quality (4–6 points) (Supplementary File 1).

**Table 2.** The main characteristics of the included articles

			Total sample size, n		Prevalence, %						
Author, year	Implementation year	Country	Cases	Controls	Cases	Controls	Study type	Diagnostic method	Cancer type	Quality score	Reference
Rudrapatna, 1997	UC	India	1029	–	3.1	–	C-S	Mic	Mixed	4	^57^
Menon, 1999	1996–1997	Malaysia	50	–	6	–	C-S	Mic	Mixed	4	^61^
Togeh, 2000	1996–1997	Iran	261	–	13.8	–	C-S	Mic	Mixed	4	^48^
Tasova, 2000	1997–1998	Turkey	206	200	6.8	2.5	C-C	Mic	HM	5	^51^
Gharavi, 2003	UC	Iran	141	70	17	11.4	C-C	Mic	HM	6	^40^
Robinson, 2006	1997–2001	Uganda	1771	–	3.5	–	C-S	Mic	Mixed	4	^64^
Monsef, 2008	2005–2006	Iran	190	–	5.8	–	C-S	Mic	Mixed	4	^45^
Idris, 2010	2008–2009	Indonesia	10	–	10	–	C-S	Mic	Mixed	7	^59^
Hazrati-Tappeh, 2011	2007–2008	Iran	101	–	7.9	–	C-S	Mic	Mixed	4	^42^
El-Mahallawy, 2011	2008–2009	Egypt	271	60	5.2	6.7	C-C	Mic	Mixed	5	^54^
Sulżyc-Bielicka, 2012	2009–2010	Poland	87	–	1.1	–	C-S	Mic	CRC	4	^63^
Al-Qobati, 2012	2011–2012	Yemen	206	–	18	–	C-S	Mic	Mixed	4	^66^
Jiménez-Cardoso, 2013	2010–2011	Mexico	77	–	2.6	–	C-S	Mol	HM	6	^62^
Durak, 2013	UC	Turkey	337	–	14.8	–	C-S	Mic	Mixed	4	^50^
El-Mahallawy, 2013	2011–2012	Egypt	89	100	14.6	16	C-C	Mic and ELISA	Mixed	6	^53^
Berenji, 2013	2008–2009	Iran	89	–	18	–	C-S	Mic	HM	5	^37^
Bora, 2016	UC	India	15	–	20	–	C-S	Mic	Mixed	5	^58^
Silva, 2016	2011–2012	Brazil	70	–	8.6	–	C-S	Mic and ELISA	Mixed	6	^56^
Abdul Hussein, 2017	2015–2016	Iraq	106	–	18.9	–	C-S	Mic	Mixed	7	^60^
Berahmat, 2017	2015–2016	Iran	132	132	3	1.5	C-C	Mic	Mixed	7	^36^
Mohammadi, 2017	2015–2016	Iran	100	–	2	–	C-S	Mic	Mixed	5	^39^
Esteghamati, 2018	2016–2017	Iran	85	–	2.4	–	C-S	Mic	Mixed	5	^38^
Jeske, 2018	UC	Brazil	73	–	16.4	–	C-S	Mic	Mixed	6	^55^
Toychiev, 2018	2015–2017	Uzbekistan	200	200	10	16	C-C	Mic	CRC	7	^65^
Taghipour, 2018	2017–2018	Iran	10	–	10	–	C-S	Mic	Mixed	5	^47^
Salehi, 2018	2016–2017	Iran	150	–	0.7	–	C-S	Mic	Mixed	4	^46^
Izadi, 2019	2015–2016	Iran	87	–	3.5	–	C-S	Mic	Mixed	7	^43^
El-Badry, 2019	2013–2015	Egypt	137	–	1.5	–	C-S	Mol	Mixed	5	^52^
Ghoyounchi, 2019	2015–2016	Iran	132	–	3	–	C-S	Mic	Mixed	6	^41^
Akgul, 2020	2016–2017	Turkey	57	90	26.3	7.8	C-C	Mol	Mixed	6	^49^
Mahmoudi, 2020	2017–2018	Iran	362	399	0	2	C-C	Mic	Mixed	7	^44^
Banihashemi, 2020	2018–2019	Iran	250	–	2	–	C-S	Mol	Mixed	5	^35^

–UC: unclear; Mic: microscopic method; Mol: molecular method; C-C: case–control study; C-S: cross-sectional study.

### Pooled prevalence of *G. duodenalis* infection in cancer patients

**Figure 2.**
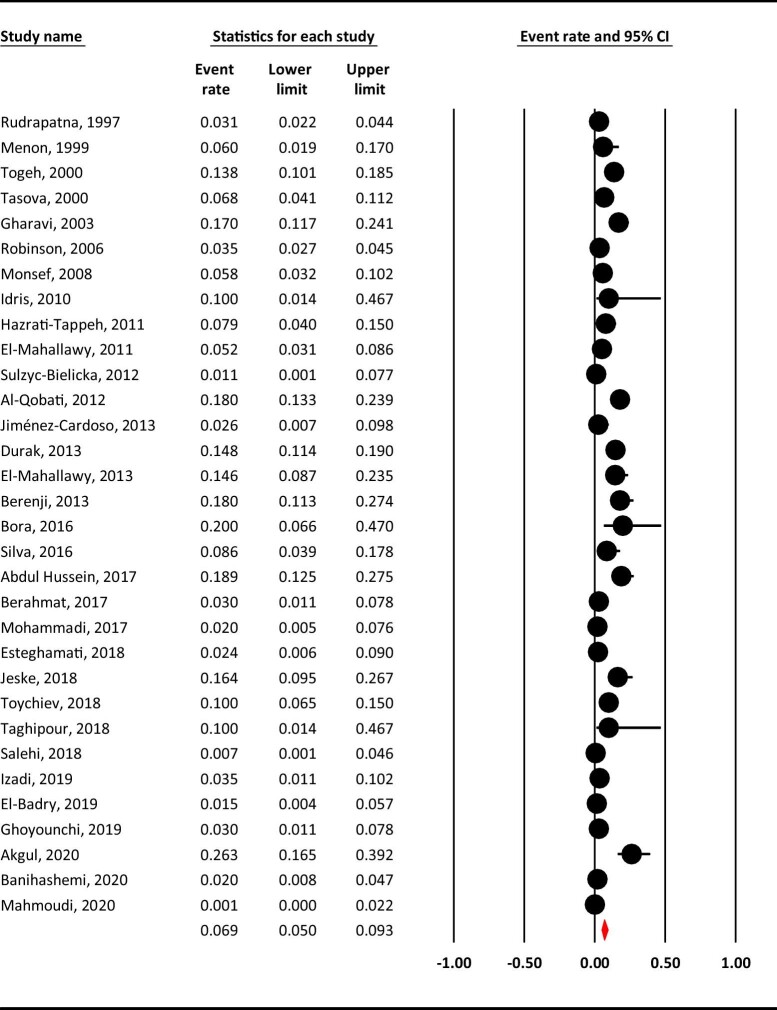
The estimated pooled prevalence of *G. duodenalis* infection in cancer patients.

The estimated weighted prevalence of *G. duodenalis* infection in cancer patients was computed to be 6.9% (95% CI 0.5 to 9.3) (Figure 2). The heterogeneity analysis illustrates that there was high-level, significant heterogeneity in our meta-analysis regarding cancer patients (Q=272.464, I^2^=88.6%, p=0.000).

### Association of cancer patients with *G. duodenalis* infection

**Figure 3.**
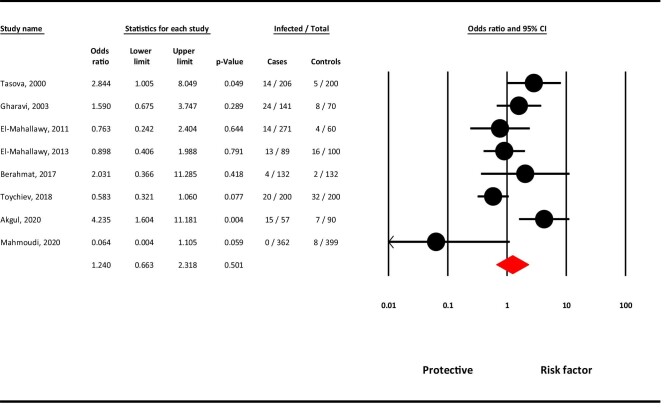
A meta-analysis of the association of cancer patients and *G. duodenalis* infection using random effects analysis.

Of the eight case–control studies conducted in four countries worldwide, the estimated pooled random effects ORs of cancer patients compared with their controls was calculated to be 1.24 (95% CI 0.66 to 2.31; p=0.501) for infection with *G. duodenalis*. In other words, cancer patients were 1.24 times more exposed to *G. duodenalis* infection than healthy controls, although this association was not statistically significant (Figure 3). Regarding case–control studies, the heterogeneity analysis showed that there was relatively high-level heterogeneity in our meta-analysis (Q=20.580, I^2^=65.9%, p=0.004).

### Sensitivity analysis

The sensitivity analysis illustrated that by ignoring each of the eight studies with ORs, there was no significant change in the final OR and, again, immunodeficiency due to cancer was not a statistically significant risk factor for *G. duodenalis* infection (Supplementary File 2).

### Subgroup analysis of *G. duodenalis* infection in different examined groups

**Figure 4.**
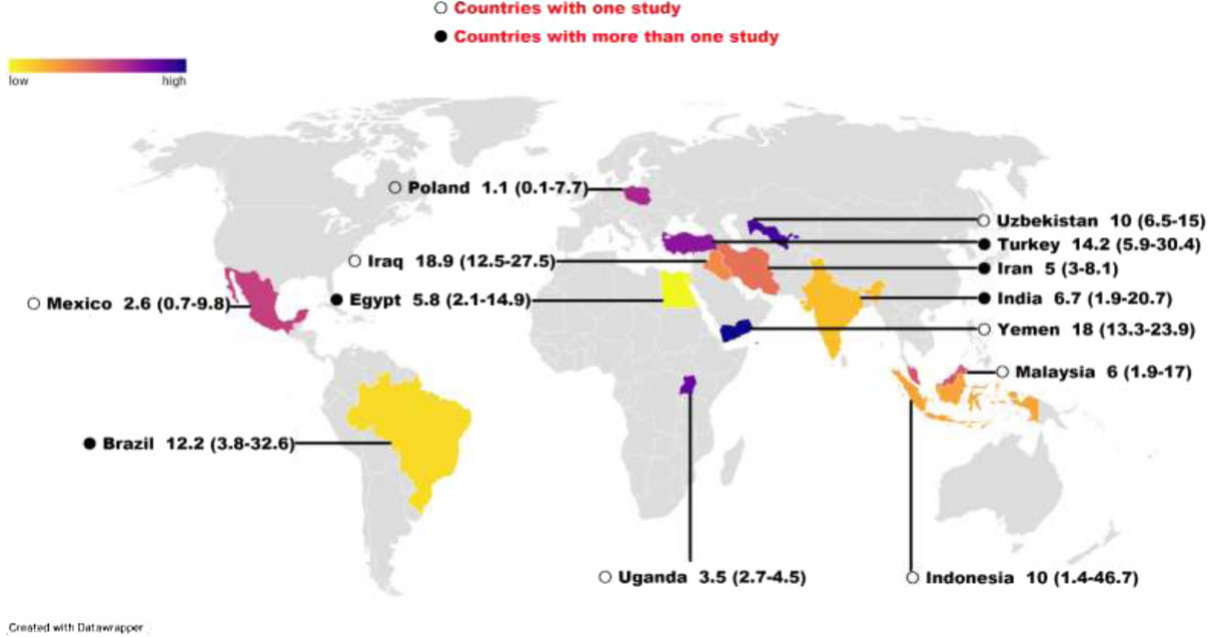
The global estimated pooled random effects prevalence of cancer patients based on each country for *G. duodenalis* infection.

The results of the subgroup analyses are shown in Table 3. The estimated pooled prevalence of giardiasis, on a country basis, is shown in Figure 4. In brief, according to continent and WHO region, the highest prevalence was reported in South America (12.20% [95% CI 3.90 to 32.10]) and the European Union region (EUR) (10.60% [95% CI 5.30 to 20.0]), respectively (p<0.001) (Supplementary Files 3 and 4). A meta-analysis of studies on cancer patients according to publication year illustrated that *G. duodenalis* infection demonstrated a decreasing trend of frequency, ranging from 17.0% (95% CI 11.70 to 24.10) between 2001 and 2005 to 5.80% (95% CI 3.70 to 8.90) between 2016 and 2020 among cancer patients worldwide (Supplementary File 5). With regard to country income, the highest and lowest prevalences were related to low-income countries (8.10% [95% CI 2.70 to 22.10]) and high-income countries (1.10% [95% CI 0.10 to 7.70]), respectively (Supplementary File 6). Regarding cancer types, the highest weighted prevalence of *G. duodenalis* infection was reported from patients with HMs (9.90% [95% CI 4.20 to 21.70]), while patients with CRC had the lowest pooled frequency of infection (5.20% [95% CI 1.30 to 19.0]) (Supplementary File 7).

**Table 3.** Subgroup analysis of the prevalence of *G. duodenalis* infection based on publication year, country income, continent, WHO region, country and cancer type

Subgroup variable	Prevalence, % (95% CI)	Heterogeneity (Q)	I^2^ (%)	p-Value
Publication year				
≤2000	6.60 (3.0 to 13.90)	40.206	92.5	<0.001
2001–2005	17.0 (11.70 to 24.10)	–	–	>0.999
2006–2010	5.10 (1.90 to 13.10)	3.441	41.9	0.179
2011–2015	9.40 (5.40 to 16.10)	35.443	80.3	<0.001
2016–2020	5.80 (3.70 to 8.90)	94.629	84.1	<0.001
Country income				
Low	8.10 (2.70 to 22.10)	65.345	98.5	<0.001
Lower-middle	6.70 (3.30 to 13.10)	39.931	87.5	<0.001
Upper-middle	7.10 (4.90 to 10.20)	118.307	81.4	<0.001
High	1.10 (0.10 to 7.70)	–	–	>0.999
Continent				
Africa	5.0 (2.20 to 11.10)	25.388	88.2	<0.001
Asia	6.60 (4.50 to 9.60)	152.255	86.9	<0.001
Europe	10.60 (4.60 to 22.50)	22.172	86.5	<0.001
North America	2.60 (0.70 to 9.80)	–	–	>0.999
South America	12.20 (3.90 to 32.10)	1.917	47.8	0.166
WHO region				
AFR	3.50 (2.70 to 4.50)	–	–	>0.999
AMR	8.50 (3.20 to 20.30)	7.124	71.9	0.028
EMR	6.30 (4.30 to 9.20)	118.138	84.8	<0.001
EUR	10.60 (5.30 to 20.0)	23.998	83.3	<0.001
SEAR	7.10 (2.50 to 18.40)	10.468	80.9	0.005
WPR	6.0 (1.90 to 17.0)	–	–	>0.999
Country				
Brazil	12.20 (3.80 to 32.60)	1.917	47.8	0.166
Egypt	5.80 (2.10 to 14.90)	13.872	85.6	0.001
India	6.70 (1.90 to 20.70)	9.414	89.4	0.002
Indonesia	10.0 (1.40 to 46.70)	–	–	>0.999
Iran	5.0 (3.0 to 8.10)	79.128	83.6	<0.001
Iraq	18.90 (12.50 to 27.50)	–	–	>0.999
Malaysia	6.0 (1.90 to 17.0)	–	–	>0.999
Mexico	2.60 (0.70 to 9.80)	–	–	>0.999
Poland	1.10 (0.10 to 7.70)	–	–	>0.999
Turkey	14.20 (5.90 to 30.40)	15.380	87	<0.001
Uganda	3.50 (2.70 to 4.50)	–	–	>0.999
Uzbekistan	10.0 (6.50 to 15.0)	–	–	>0.999
Yemen	18.0 (13.30 to 23.90)	–	–	>0.999
Cancer type				
CRC	5.20 (1.30 to 19.0)	4.763	79	0.029
HM	9.90 (4.20 to 21.70)	16.375	81.7	0.001
Mixed	6.50 (4.50 to 9.30)	240.634	89.6	<0.001

### Meta-regression

**Figure 5.**
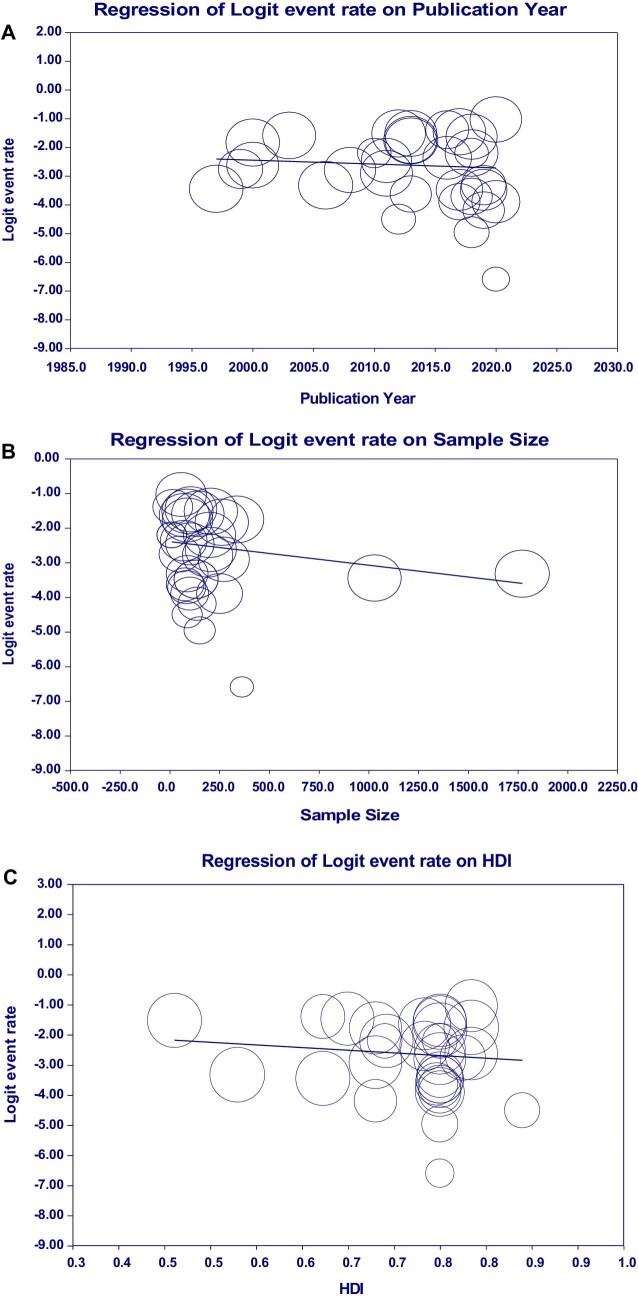
The meta-regression shows an absence of a statistically significant association between the prevalence of *G. duodenalis* infection in cancer patients and quantitative variables such as (A) publication year, (B) sample size and (C) HDI.

Our meta-regression results did not report a statistically significant association between the prevalence of *G. duodenalis* infection in cancer patients and quantitative variables such as publication year, sample size and HDI. Therefore the year of study (regression coefficient −0.0135, p=0.578), sample size (regression coefficient −0.0007, p=0.074) and HDI (regression coefficient −1.6263, p=0.419) were not considered as a cause of variability in the results of *Giardia* infection rate in cancer patients (Figure 5).

### Publication bias

**Figure 6.**
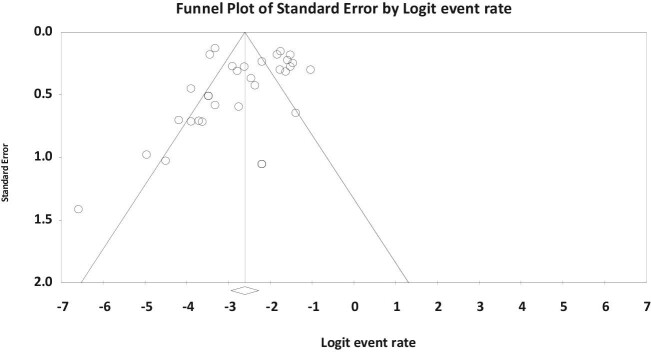
Funnel plot showing the absence of publication bias among the included studies (p=0.221).

There was no significant publication bias in the present systematic review and meta-analysis (p=0.221) (Figure 6).

## Discussion

A prevalence rate of 0.4–30% is estimated for *Giardia* infection in immunocompetent hosts,^10,11^ while there is no available information regarding the total prevalence and likely pathogenicity of *G. duodenalis* in immunocompromised people, especially in cancer patients. Therefore we conducted the present systematic review and meta-analysis to elucidate the prevalence and risk factors of *G. duodenalis* infection among cancer patients worldwide. Also, the association of immunodeficiency status with the parasitic infection was evaluated by estimation of a pooled OR derived from case–control studies.

A relatively moderate worldwide prevalence (6.9%) of *Giardia* infection in cancer patients was the principal finding of the present review. Moreover, cancer patients were shown to be 1.24-fold more susceptible and were at a higher risk of infection, which should alert physicians to the possible consequences. Due to the lack of previous meta-analyses on the pooled prevalence and/or weighted OR estimation of intestinal parasites in cancer patients, accurate inference and comparison of the results are problematic. In a similar study on *Blastocystis*, a zoonotic intestinal agent, a weighted frequency of 9% was obtained in cancer patients.^67^ This higher prevalence in such a susceptible group compared with *Giardia* infection may be justified by the fact that *Blastocystis* is recognized as the most common parasitic agent reported in human faecal samples.^68–70^ Also, Kalantari et al.^71^ reported that there is a positive association between *Cryptosporidium* infection and cancer (OR 3.3 [95% CI 2.18 to 4.98]), consistent with our findings. Their results revealed that *Cryptosporidium* is a highly opportunistic apicomplexan parasite and impaired immunity is a strong risk factor for this infection. However, our review and the report by Kalantari et al.^71^ were based on a limited number of investigations, hence more extensive studies are required to yield a more reasonable inference. Regarding publication year, no specific trend was observed for *Giardia* infection; accordingly, the prevalence of infection was highest between 2001 and 2005, followed by a rapid decline until 2010. Also, the presence of a 4- to 5-y gap between the implementation and publication years complicates the true inference of the results.^37,56,64^

The estimated pooled prevalence of *Giardia* infection varied among geographical regions, with the highest being reported from the EUR and Iraq, whereas the lowest prevalence was reported from the WHO African (AFR) region and Mexico. However, evaluation of the prevalence based on WHO regions is not so reliable, as countries in a particular region may demonstrate different parameters regarding geographical location or distance. In terms of continents, South America and North America showed the highest and lowest prevalence rates, respectively. However, most of the studies on *Giardia* prevalence in cancer patients were related to the Asian continent and there are very limited reports from other continents. The different weighted frequency of the infection among global regions results from the number of studies, geographical differences, treatment stage at the time of sampling and the sensitivity of diagnostic methods. In addition, the greater was a country’s income, the lower was the prevalence of *Giardia* infection; accordingly, the highest pooled prevalence rates were in low-income countries. Interestingly, the only high-income country included in our review was Poland,^63^ which does not appropriately represent the true prevalence of the infection in a given subgroup. At first glance, the weighted prevalence of infection in African nations was expected to be equal to that in low-income countries, while a closer look showed that since some Asian countries, for example, Yemen,^66^ are included in the low-income group, the prevalence of giardiasis in African nations varies from low-income ones. In addition, some African countries such as Egypt^52–54^ are not included among low-income nations, which causes a difference in the weighted prevalence.

Interestingly, the weighted prevalence of giardiasis was higher among patients suffering from HMs compared with CRC patients as well as those individuals with mixed cancers. The same locale for both CRC and *Giardia* may direct one's mind to the higher prevalence of infection among CRC patients, but this information was not supported by obtained evidence in the present review. Generally culture and microscopic methods are considered as the gold standard diagnostic technique for giardiasis.^18^ However, increasing utilization of molecular tests demonstrates that the direct method of DNA extraction from stool samples is very sensitive for accurate diagnosis of this parasitic infection.^72^ Certainly the limited number of studies and different sensitivities and specificities of methods have caused bias,^73–75^ and the method-based prevalence was not provided in the present review due to the unreliability of data. There was no significant publication bias (p=0.221) based on the included papers in the present review, indicating that published studies are a representative sample of the available evidence.

In total, the present systematic review and meta-analysis showed some strengths: evaluation of the pooled frequency of the *G. duodenalis* infection among 7024 cancer patients from 13 different countries on five continents, estimation of pooled random effects ORs of *Giardia* infection in cancer patients compared with control groups and subgroup analysis regarding publication year, continent, country, WHO region, country income and cancer type. However, the present review had some limitations: a lack of prevalence studies in several countries, the absence of sufficient molecular studies investigating the prevalence of *Giardia* infection, not including various risk factors such as age and sex in some studies, including some studies with very small sample sizes and a lack of adequate studies on the prevalence of *Giardia* infection in patients with various cancer types. The lack of studies obviously biased our results; for example, the global weighted OR reported here was only inferred from eight studies in four different countries. Furthermore, the pooled prevalence of the infection estimated in the present review (6.9%) was approximately based on the microscopic method. This was not a surprising finding, since *Giardia* can be easily detected by its unique morphology in infected stool specimens. However, with the increasing use of molecular studies, more aspects of *Giardia* epidemiology in cancer patients can be identified. Inevitably, these limitations would have a substantial impact on the prevalence status of giardiasis in cancer patients that should not be ignored. With all these limitations, the present work tried to show a clear estimate of *Giardia* infection prevalence in cancer patients based on the current status of science, which may be elucidated in the near future by the implementation of extensive research.

## Conclusions

To the best of our knowledge this is the first systematic review and meta-analysis showing a general overview of *G. duodenalis* infection prevalence and associated risk factors among cancer patients globally. The results indicated a mild prevalence in such at-risk patients, although based on the weighted OR, the immunodeficiency status of the examined hosts was not a statistically significant risk factor for *Giardia* infection. Our results demonstrated that the immunodeficiency status of cancer patients is a possible risk factor for acquiring *Giardia* infection, which requires strict preventive measures. Altogether, with the limited number of studies, it was not possible to accurately investigate the association between the prevalence of *Giardia* infection and a patient's immunodeficiency status. Achieving this goal will require more extensive cohort and case–control studies, particularly in neglected areas of the world.

## Supplementary Material

ihab026_Supplemental_Files

## Data Availability

The data underlying this article are available in the article and in its online supplementary material.
