# Supplementary material for: Global epidemiology of Giardia duodenalis infection in cancer patients: a systematic review and meta-analysis
Source: Int Health. 2021 May 22;14(1):5–17. doi: 10.1093/inthealth/ihab026 (PMC8769951; doi:10.1093/inthealth/ihab026)
Supplement: ihab026_Supplemental_Files [file ihab026_supplemental_files.zip › Suppl_file_4.docx]

**Supplementary File. 4.** The pooled random-effects prevalence of *Giardia duodenalis* infection in cancer patients based on WHO regions
